# [Corrigendum] A regulation loop between Nrf1α and MRTF-A controls migration and invasion in MDA-MB-231 breast cancer cells

**DOI:** 10.3892/ijmm.2024.5442

**Published:** 2024-10-21

**Authors:** Yao Xu, Ying Luo, Chen Liang, Weibing Xing, Tongcun Zhang

Int J Mol Med 42: 2459-2468, 2018; DOI: 10.3892/ijmm.2018.3816

Subsequently to the publication of this article, an interested reader drew to the authors' attention that the Control and Nrf1α data panels in Fig. 1G on p. 2463 contained overlapping data, such that these data, which were intended to show the results from differently performed experiments, had apparently been derived from the same original source. Upon examining their original data, the authors realized that the image for the Control experiment was selected incorrectly for this figure.

In rectifying this error, the authors have chosen to show the data from one of their repeated experiments for Fig. 1G, and the revised version of this figure is shown on the next page. They can confirm that the replacement of these data in this corrigendum does not significantly affect the conclusions reported in the study. The authors are grateful to the Editor of *International Journal of Molecular Medicine* for allowing them the opportunity to publish this corrigendum, and wish to apologize to readership for any inconvenience caused.

## Figures and Tables

**Figure 1:**
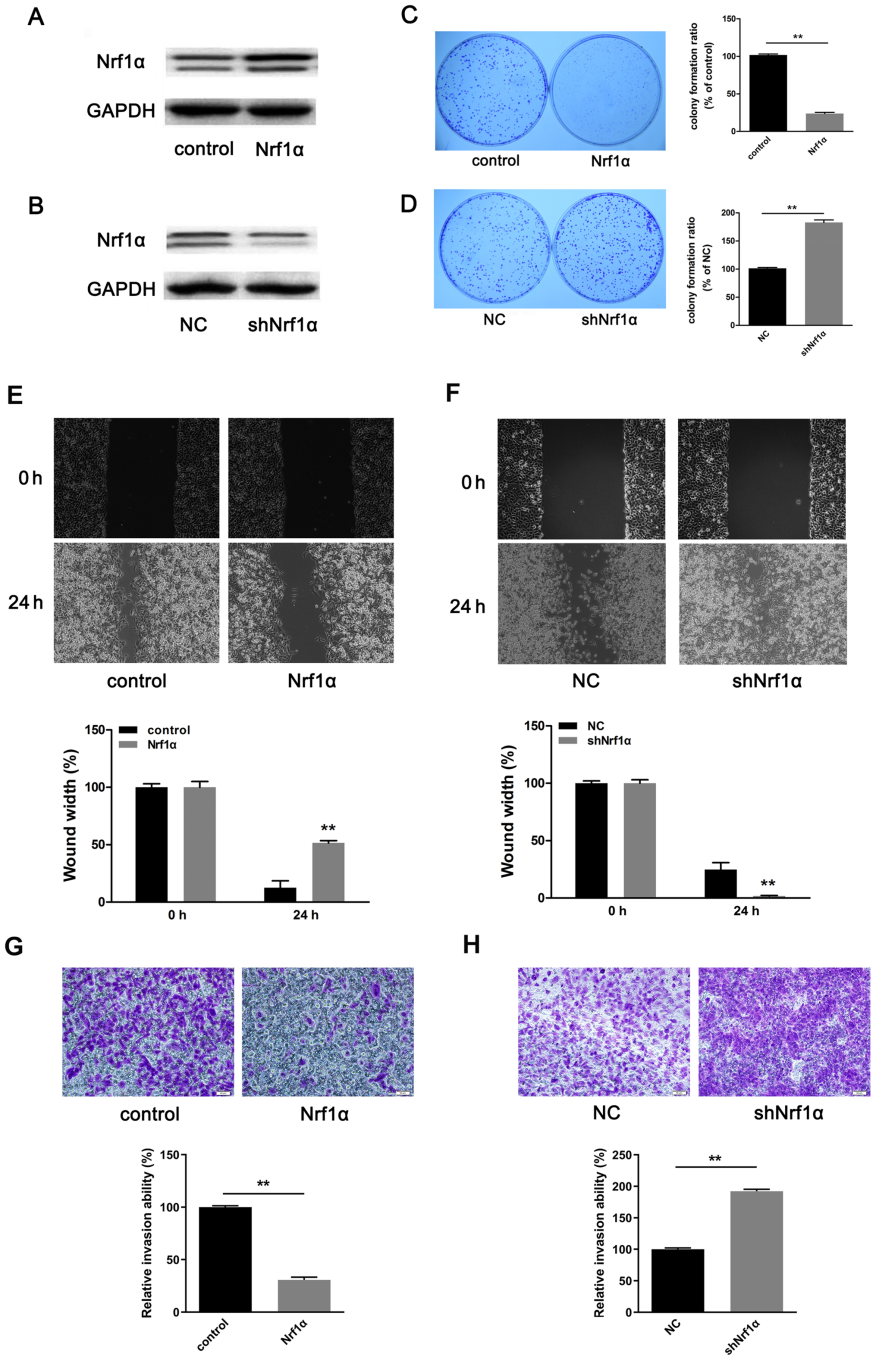
Nrf1α inhibits migration and invasion in MDA-MB-231 cells. (A) Western blotting assays were performed to quantitatively measure the protein levels of Nrf1α following overexpression or (B) shRNA knockdown. GAPDH expression was used as an internal control to show equal loading of the protein samples. (C) Colony formation assay was performed to determine the proliferation of MDA-MB-231 cells following Nrf1α overexpression or (D) shRNA knockdown. (E) Cell migration was detected by wound healing assay following Nrf1α overexpression or (F) shRNA knockdown. (G) Transwell invasion assay for Nrf1α or (H) shNrf1α-transduced MDA-MB-231 cells. n≥3. ^**^P<0.01 compared with control. Nrf1α, nuclear factor erythroid 2-like 1; sh, short hairpin; NC, negative control.

